# Innovative Inclusion Complexes Clotrimazole: Hydroxypropyl-β-Cyclodextrin-Modified Polyurethane Networks as Carriers for Slow Drug Delivery †

**DOI:** 10.3390/biomedicines14030666

**Published:** 2026-03-14

**Authors:** Suzana M. Cakić, Snežana S. Ilić-Stojanović, Ljubiša B. Nikolić, Vesna D. Nikolić, Ivan S. Ristić, Gordana S. Marković, Nada Č. Nikolić

**Affiliations:** 1 Faculty of Technology, University of Nis, Bulevar Oslobodjenja 124, 16000 Leskovac, Serbia; cakics@tf.ni.ac.rs (S.M.C.);; 2 Faculty of Technology, University of Novi Sad, Bulevar car Lazara 1, 21000 Novi Sad, Serbia; 3 Tigar A.D., Nikole Pašića 213, 18300 Pirot, Serbia

**Keywords:** inclusion complex, hydroxypropyl-β-cyclodextrin, polyurethane, clotrimazole, thermal analysis, x-ray diffractometry, SEM, HPLC, slow release

## Abstract

**Background/Objectives**: Inclusion complexes among drugs and cyclodextrin-modified polymers are a topic of recent interest in pharmaceutical research and industry as they might expand the solubility, bioavailability, and stability of the guest molecules. Polyurethanes derived from cyclodextrins show some biomedical applications. In this study, two cross-linked polyurethane networks based on hydroxypropyl-β-cyclodextrin (HPβCD) and polyethylene glycols (PEG 2000 or PEG 6000) were synthesized with NCO/OH molar ratio 4.3 and 6.3 by the typical two-step polymerization method. **Methods**: Inclusion complexes of clotrimazole (CLOT) with two HP*β*CD-modified polyurethane networks and their corresponding physical mixtures were prepared using kneading methods and physical mixing in a 1:6 weight ratio of CLOT:HPβCD. **Results**: Obtained prepolymers, previously end-capped with isocyanate groups forming urethane links with HPβCD, which were confirmed by FTIR analysis. TGA results indicate a slight increase in thermal stability of the prepared complexes. The characteristic endothermic peak of the CLOT at around 145.90 °C did not appear in the DSC curve of the drug-loaded inclusion complexes. The XRD patterns of physical mixtures showed specific peaks corresponding to pure clotrimazole. SEM micrographs confirmed an elliptical/spherical- and plate-shaped particles without phase segregation, indirectly confirming that CLOT is not separately present due to inclusion into HPβCD and entrapment into polyurethane networks. Novel complexes PUR2/HPβCD-CLOT-IC and PUR3/HPβCD-CLOT-IC were applied as drug carriers, and diffusion-controlled kinetics of CLOT release were best described using Higuchi model. **Conclusions**: The obtained in vitro results showed surprisingly slow/prolonged clotrimazole release from modified polyurethane networks due to the significant influence of NCO/OH molar ratio and the chosen polyol soft segments chain length with potential in vivo applications.

## 1. Introduction

Slow drug release from polymer matrices has garnered attention among researchers due to its target-specificity and the economics of the slow-release strategy.

The slow release of drugs from biocompatible and biodegradable polymer matrices has gained worldwide attention among researchers due to the realization of target-specific controlled delivery of the active ingredient in vivo, as well as the accruing economics of the slow-release technology [1]. Polyurethanes (PURs) are an important group of materials which have biomedical applications such as tissue engineering, orthopedic implants, transdermal patches, and drug delivery carriers. The kinetics of swelling and the sorption performance were observed by Renuka and Sonal [2] for the interpenetrating polymer networks of biodegradable polyurethanes with carbohydrate cross-linkers. They are synthesized biodegradable PURs by using carbohydrates such as glucose (monosaccharide), sucrose (disaccharide) or starch (polysaccharide) as a cross-linker and by varying the NCO/OH and diol/triol/molar ratios.

**Figure 1:**
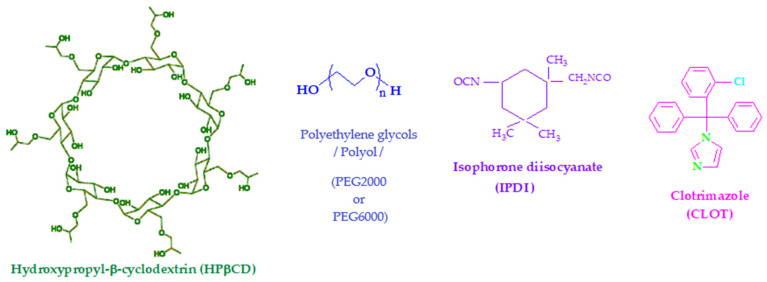
Chemical structure depiction of main reactants: 2-hydroxypropyl-β-cyclodextrin (HPβCD), polyethylene glycols/polyol (PEG 2000 or PEG 6000), isophorone diisocyanate (IPDI), and clotrimazole (CLOT).

Currently, chemical modifications of natural carbohydrates as well as cyclodextrins (CDs) have been extensively reported [3]. They are cyclic oligosaccharides containing six (α-cyclodextrin), seven (β-cyclodextrin), or eight (γ-cyclodextrin) glucopyranose units linked by α-(1,4) glycosidic bonds. The structure of CDs molecules resembles truncated cones with the secondary hydroxyl groups located at the wider edge of the ring and the primary groups on the narrower edge. Therefore, CDs have relatively hydrophobic cavities, while their outer surfaces are hydrophilic. These CDs can form reversible host-guest inclusion complexes with a wide variety of inorganic and organic molecules in aqueous solution [4,5]. Hydroxypropyl-β-cyclodextrin (Figure 1), as an alternative with lower toxicity than α-, β-, or γ-CDs, can improve the water solubility of lipophilic compounds, bioavailability, and stability, as well as enable better formulations [6,7].

Polymeric nanoparticles encompass a variety of particles ranging in size from 1 nm to 1000 nm and could be classified as nanocapsules (drug dispersed in a polymer matrix) or nanospheres (drug dispersed in a liquid core encapsulated by a polymer membrane) [4,8]. The polymers used must be biocompatible, easy to obtain, stable, with appropriate biodegradation kinetics, and hypotoxic, while maintaining their properties for a limited time in vivo and with slow degradation of soluble compounds [9,10]. Polymeric nanoparticles show improved encapsulation efficiency compared to other nanoparticles, as they can be controlled by the characteristics of the components that make up the formulation and the method of preparation [11,12,13,14]. The polymer component of the nanoparticle provides additional steric stability and protection against potential changes.

The aggregation of α-, β-, and γ-CDs is a recognized phenomenon as they form self-assembled aggregates in concentrated solutions and bind together by forming a hydrogen bond network, so-called “poly-CD” with optimal configuration “head-to-head/tail-to-tail” orientation of the neighboring CD rings [15,16]. Their mixing with the poly(propylene glycol) or polyethylene glycol (PEG) causes the reversible threading of the CDs molecules along a single polymeric chain, named polypseudorotaxane [15,17,18]. Chemical modification or polymerization of the CD rings is the other possibility that can form CD-based nanostructures. CDs are also used for the formation of different nano-scale drug delivery systems like CD polymers and nanosponges [16]. The 2,3,6-hydroxyl groups of the cyclodextrin ring show different reactivity and can be modified (2,6-OH is the most reactive) [19], which can help their extensive pharmaceutical applications. The main methods for the synthesis of modified CDs are deprotonation, dehydration and condensation. The condensation is the reaction of CDs directly with diisocyanate as a bifunctional linker.

PURs containing CDs have been used to combine synergistically polymer characteristics and inclusion properties of CDs. The lack of good thermal properties, poor solubility, and difficult processing are serious limitations of these materials when they are used alone, but the inclusion of cyclodextrins in the structure of PURs can result in obtaining biodegradable materials with improved mechanical and thermal properties. Therefore, cross-linked polyurethane networks can be obtained by reacting hydroxyl groups of CDs with a diisocyanate, such as isophorone diisocyanate (IPDI, Figure 1), leading to the formation of the urethane linkage (NHCOO). However, few papers are devoted to studying the inclusion capacity of cross-linked CDs into the polyurethane network for pharmaceutical purposes. Cross-linked copolymers based on β-cyclodextrin, polyethylene glycol (PEG, Figure 1) and 4,4′-diphenylmethane diisocyanate were prepared as polymeric solid–solid phase change materials which may be applied in thermal energy storage and temperature control because they exhibit heat storage and release properties due to the phase transition temperature [20]. de Araújo et al. obtained cross-linked polyurethanes with βCD and HPβCD, PEG 400, PEG 1500 or PEG 4000, and TDI, characterized them using FTIR, XRD and TGA methods [5]. They were used as carriers for nifedipine delivery and achieved 80% of nifedipine released during 9.42 h in a slightly acidic solution (pH 6.8). Polyethylene glycol and oligocaprolactone-modified cyclodextrin were prepared by polyaddition cross-linking using isophorone diisocyanate [16]. The cross-linked material was characterized by FTIR, MALDI MS, SEM, TG, DTG, DSC, and dynamic rheology methods. Thermal water swelling and hydrolytic degradation under alkaline conditions revealed the connectivity of the polymer network and the influence of the amount of cross-linked cyclodextrin on the hydrogel properties. Hydrolytically degradable polymer networks with hydrophilic character of levofloxacin as a guest molecule in the cyclodextrin cavity were bonded through physical interactions. A burst release of levofloxacin was observed in the first 50 min (up to 60% of the drug loaded), followed by a sustained release over the next few hours [16].

Clotrimazole (CLOT) (Figure 1), an imidazole derivative, is an antifungal medication that prevents mycotic infections of the gastrointestinal and urinary tract and skin. It has also been reported as an anti-cancer drug based on its ability to inhibit mitochondrial-bound glycolytic enzymes and calmodulin, thereby affording cancer death by autophagy. It is available in numerous conventional dosage forms such as tablets, ointments, creams, vaginal suppositories and solutions, but these conventional dosage forms have not been known to afford slow release of CLOT due to the relatively short residence time [1]. This has resulted in an impairment of the therapeutic efficacy of the drug, necessitating multiple administrations. It is generally safe and poses no risk of acute intoxication when administered topically because of minimal absorption. The primary toxicity risks could be associated with oral lozenges, which can cause gastrointestinal distress (nausea, vomiting), elevated liver enzymes (up to 15% of cases), and potential skin irritation. When clotrimazole is applied topically and locally, toxic effects (i.e., erythema, skin rash, edema, pruritus, urticaria, pelvic cramps, itching, vulva and vagina irritation) may occur due to overdosing [21,22]. Doses up to 100 times the human dose in animal studies were embryotoxic in mice and rats. Otherwise, CLOT is a lipophilic compound with poor aqueous solubility (5.5 μmol/L) and is practically insoluble, requiring effective formulations based on lipid carriers, surfactants, or CDs complexes to enhance its bioavailability [23].

This work aimed to expand the knowledge about cross-linked polyurethane networks based on CDs for pharmaceutical use as a controlled drug delivery system. For this purpose, polyurethanes (PURs) were synthesized by using hydroxypropyl-β-cyclodextrin (HPβCD) as a cross-linker of alicyclic isophorone diisocyanate (IPDI) and two polyethylene glycols (PEG 2000 and PEG 6000). The specific goal of this work was to investigate the influence of different PEG chain lengths, which constitute the soft segments of PUR, by varying the NCO/OH molar ratio on the structural characteristics of the polymer networks, and in particular on the drug release profile and kinetics. This study reported direct, simple, and effective inclusion of CLOT in obtained cross-linked HPβCD-modified polyurethane networks with PEG 2000 or PEG 6000 (PUR2/HPβCD or PUR3/HPβCD, respectively), including their cross-linking, characterization, and application as carriers. In vitro drug release studies were conducted to determine the drug release pattern from two cross-linked HPβCD-modified polyurethane networks.

Based on available data, the potential of such HPβCD-modified cross-linked polyurethane networks for usage as a carrier for prolonged clotrimazole release has not been reported in the literature.

## 2. Materials and Methods

### 2.1. Reagents

Polyethylene glycols for synthesis (PEG 2000, average M_w_~2000 g/mol, PEG 6000, average M_w_~6000 g/mol, molecular biology grade) obtained from Sigma Aldrich, Burlington, MA, USA, were dried under vacuum at 80 °C for 12 h before being used as polyols. Hydroxypropyl-β-cyclodextrin 97% (HPβCD), average M_w_~1540 g/mol (Figure 1), purchased from Aldrich chemistry (Sigma-Aldrich, Burlington, MA, USA), was used as received.

Clotrimazole, 98.5–100.5% (dry basis) powder (M_w_ = 344.84 g/mol, aqueous solubility 5.5 μmol/L), as depicted in Figure 1, was purchased from Sigma Chemicals (Steinheim, Germany).

Isophorone diisocyanate (M_w_ = 222.28 g/mol, 98wt% purity, from Sigma Aldrich, Burlington, MA, USA) (IPDI) (Figure 1) and dibutyltin dilaurate 95% (from Bayer, Leverkusen, Germany) (DBTDL) were used without further purification. The following compounds were obtained from commercial suppliers and used as received: dimethyl formamide, 99.8% (DMF) (Merk-Schuchardt, Hohenbrunn, Germany) and methanol, >95% (Zdravlje, Leskovac, Serbia).

### 2.2. Synthesis of HPβCD-Modified Polyurethane Networks

The biodegradable waterborne HPβCD-modified polyurethane networks were prepared using a two-step polymerization process as described in a previously published paper [24]. Two prepolymer solutions were obtained by mixing PEG 2000 or PEG 6000 polyols (2.00 g), isophorone diisocyanate (2.22 g), and dibutyltin dilaurate (0.30 g) with dimethyl formamide as a solvent (30 mL) in a glass round flask with a four-neck, linked to a thermometer, reflux condenser, and nitrogen gas inlet (40 mL/min flow rate). These solutions were mixed and mechanically stirred for 90 min (180–200 rpm) at 50 °C to get a homogeneous mixture. In the next step, HPβCD (1.93 g) was dissolved in dimethyl formamide (30 mL), and dibutyltin dilaurate (0.30 g) was added to each prepolymer mixture. Obtained mixtures were reacted at 70 °C for 8 h. To precipitate the formed cross-linked HPβCD-modified polyurethane networks, methanol was added in excess, and they were washed with water and acetone and finally dried at 80 °C for 24 h. Both products—cross-linked HPβCD-modified polyurethane networks with PEG 2000 and PEG 6000—were labeled as PUR2HPβCD and PUR3HPβCD, respectively. The NCO/OH molar ratios in samples PUR2HPβCD and PUR3HPβCD were 4.3 and 6.3 [24,25].

### 2.3. Inclusion Complexes Clotrimazole: HPβCD-Modified Polyurethane Networks and Physical Mixtures Obtaining

The host-guest inclusion complexes PUR2/HPβCD-CLOT-IC and PUR3/HPβCD-CLOT-IC were obtained by the kneading method. Slight quantities of ethanol were added to the solid mixtures of CLOT and PUR2/HPβCD or PUR3/HPβCD and kneaded in a mortar for 60 min to obtain paste-like complexes (PUR2/HPβCD-CLOT-IC and PUR3/HPβCD-CLOT-IC), which were dried in a vacuum oven at 30 °C for 24 h. The maximum available amount of clotrimazole added to the inclusion complexes and physical mixtures was 8.3 mg/g.

Physical mixtures PUR2/HPβCD/-CLOT-PM and PUR3/HPβCD/-CLOT-PM were prepared from weighted CLOT (16.6% *w*/*w*) and cross-linked PUR2HPβCD- or PUR3HPβCD-modified polyurethane networks by blending for 15 min homogeneously in a mortar.

Obtained inclusion complexes (PUR2/HPβCD-CLOT-IC and PUR3/HPβCD-CLOT-IC) and physical mixtures (PUR2/HPβCD/-CLOT-PM and PUR3/HPβCD/-CLOT-PM) containing clotrimazole were pulverized.

### 2.4. Characterization of Inclusion Complexes PUR2/HPβCD-CLOT-IC and PUR3/HPβCD-CLOT-IC

Fourier transform infrared (FTIR) spectra were recorded on a BOMEM MB-100 FTIR MB-series spectrometer (Hartmann & Braun, Québec City, QC, Canada), in the wave band range from 400 to 4000 cm^−1^. FTIR spectra were recorded by the KBr pelleting technique at a resolution of 4 cm^−1^ for 32 scans and processed with Win-Bomem Easy software, version 3.01C Level II Driver Version 1.08–WBE08.

The thermal stability of samples was examined by applying differential thermal analysis (DTA) and nonisothermal thermogravimetric analysis (TGA) with a SETERAM SETSYS Evolution-1750 instrument, Caluire, France. The measurements were conducted at a heating rate of 20 °C/min, in a nitrogen atmosphere (a flow rate of 20 cm^3^/min) and in temperature range from 30 to 600 °C.

Thermogravimetric analysis (TGA) was performed using TA Instruments Q500, New Castle, DE, USA. Analyzed samples (~5 mg) were heated from 30 °C to 500 °C at a 20 °C/min heating rate in a nitrogen atmosphere (60 cm^3^/min gas flow rate). The temperature differences and weight loss during the heating period were recorded as a function of temperature.

Differential scanning calorimetry (DSC) was carried out on a TA Instruments, Q20, New Castle, DE, USA (software TA Universal Analysis, 2000, version 4.5A (Build 4.5.0.5, Copyright© 1998–207 TA Instruments—Waters LLC)). Thermal changes in samples (2–5 mg) were examined in the temperature interval 25–350 °C, at a heating rate of 10 °C/min. The melting enthalpy of indium was used in the heat flow calibration.

Wide-angle X-ray scattering measurements were carried out at room temperature with SAXSess mc^2^ instrument (Anton Paar, Graz, Austria) using monochromatized CuK_α_ radiation with wavelength, λ = 0.1542 nm generated by GeniX microfocus X-ray source (Xenocs SAS, Grenoble, France) with a power of 50 W. PerkinElmer Cyclone^®^ Plus Storage Phosphor System (PerkinElmer, Inc., Shelton, CT, USA) was used as the X-ray detector. The measurements were performed in transmission mode. The crystallinity degree was calculated as the ratio of crystallinity peak area and the sum of peak areas in the 2θ range 5–25°.

Scanning electron microscopy (SEM) was applied to observe the surface structures of the CLOT, synthesized PURs with HPβCD, and these inclusion complexes with CLOT. The samples were sprayed by an alloy of gold and palladium (85/15%) under a vacuum in a Fine Coat JEOL JFC-1100 Ion Sputter (JEOL Ltd., Tokyo, Japan). The metalized samples were scanned with a JEOL Scanning Electron Microscope JSM-5300 (JEOL Ltd., Tokyo, Japan).

### 2.5. Loading Efficiency and In Vitro Clotrimazole Release Study from Inclusion Complexes PUR2/HPβCD-CLOT-IC and PUR3/HPβCD-CLOT-IC

The release of CLOT from cross-linked HPβCD-modified polyurethane networks with PEG 2000 and PEG 6000 was analyzed by reverse-phase high-performance liquid chromatography (HPLC) on an Agilent 1100 Series HPLC device with a DAD 1200 Series detector (Waldborn, Fulda, Germany). A Zorbax Eclipse XDB-C18 column (250 mm length × 4.6 mm internal diameter, 5 μm particle size) (Agilent Technologies, Inc., Santa Clara, CA, USA) was used (25 °C). The mobile phase was methanol with a flow rate of 1 mL/min, the volume of injected samples was 20 μL, and the wavelength of detection was set to 230 nm. Data was recorded and processed on Agilent ChemStation software ChemStation for LC 3D systems Rev B02.01–SR1.

The calibration curve was obtained from a stock solution of CLOT dissolved in methanol at a concentration of 500 μg/mL. The stock solution was then diluted with the mobile phase. The retention time of CLOT was approximately 4.5 min. Valid concentrations for quantification of the CLOT in methanol were in the range of 1–200 μg/mL. The equation of the calibration curve was A = 90.04 + 25.3 *c* (*R*^2^ = 0.9984), where A is the peak area (mA·s), and *c* is the content of clotrimazole (μg/mL).

CLOT loading efficiency was determined using the HPLC method and the calibration curve equation after extensive extraction of the drug loaded in methanol. The loading efficiency (%LE) of CLOT was calculated using the following equation [26]:

(1) $$\%\mathrm{LE}\;=\;\frac{W_{drug}}{W_{polymer}+W_{drug}}\times 100$$

where *W_drug_* is the weight of the loaded drug/CLOT, and *W_polymer_* is the weight of the cross-linked HPβCD-modified polyurethane network. The calculated CLOT loading efficiency was 16.60% and 16.64% for PUR2/HPβCD-CLOT-IC and PUR3/HPβCD-CLOT-IC, respectively.

The in vitro release of clotrimazole from the inclusion complexes PUR2/HPβCD-CLOT-IC- and PUR3/HPβCD-CLOT-IC-modified polyurethane networks was performed by preparing a release medium (pH 6.8). The 10 mL aqueous solutions of inclusion complexes of clotrimazole at 0.5% (*w*/*v*) were mixed with 1 mL of Tween 20 (0.15%). The addition of polysorbate nonionic surfactant could improve the poor water solubility of clotrimazole and achieve uniform dispersion by preventing mixture aggregation in the course of stirring. The solvent systems were kept at a thermostatically controlled temperature of 37 ± 0.5 °C and stirred at 100 rpm to simulate in vivo conditions. At predetermined intervals, aliquots of 0.25 mL dissolution medium were taken and then diluted by adding 0.75 mL mobile phase. The release medium was filtered through a 0.45 μm syringe-driven membrane filter. The filtrates were assayed to determine the amount of dissolved drug using the HPLC conditions mentioned previously, except for the volume of injection (20 μL).

CLOT release kinetics from the inclusion complexes PUR2/HPβCD-CLOT-IC-and PUR3/HPβCD-CLOT-IC-modified polyurethane networks were estimated using diverse mathematical models (zero-order, first-order, Higuchi, Korsmeyer–Peppas, and Hixson–Crowell), using the DDSolver package for the Microsoft Excel add-in, v2.0 application.

## 3. Results and Discussion

### 3.1. Inclusion Complexes Clotrimazole: HPβCD-Modified Polyurethane Networks

**Figure 2:**
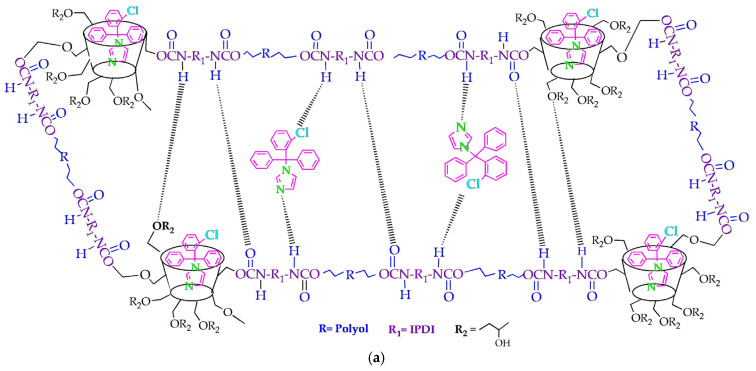

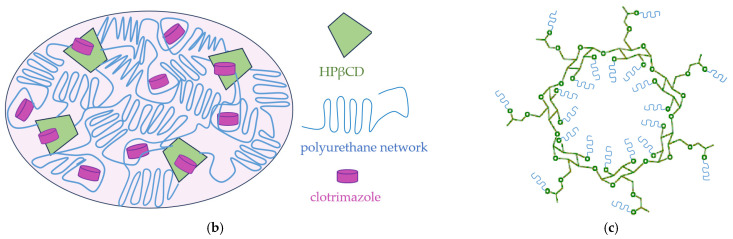
(**a**) A possible structure of a segment of the inclusion complexes clotrimazole: HPβCD-modified polyurethane networks with probable intramolecular interactions, (**b**) a representation of probable cross-linking within the nanoglobule, and (**c**) HPβCD with possible modifications for cross-linking.

A possible schematic representation of a segment of the obtained inclusion complexes clotrimazole: HP*β*CD-modified polyurethane networks, prepared by a known two-step polymerization process using the main reactants polyols (PEG2000 or PEG6000), IPDI, and HP*β*CD with the inclusion of CLOT, as described earlier [25], is depicted in Figure 2a. The obtained products are in the form of three-dimensionally cross-linked polymer nanoglobules, containing a large number of HPβCD molecules (31.48% HPβCD related to total reactant mass), which act as multifunctional cross-linking nodes and are interconnected/cross-linked via PEG segments in a possibly random/statistical arrangement (Figure 2b). Each of the HPβCD molecules in its structure has 7 glucopyranose units and 21 hydroxyl groups of different reactivity (7 primary and 14 secondary), so theoretically it could form multiple urethane bonds and represent a multifunctional cross-linker (Figure 2c). At the same time, these OH groups of HPβCD have different reactivity, which could reduce the probability that they will all react by forming covalent bonds.

### 3.2. Structural Identification of Cross-Linked HPβCD-Modified Polyurethane Networks

For an investigation of the molecular interactions of the cross-linked HPβCD-modified polyurethane networks, CLOT in physical mixtures, and the inclusion complexes PUR2/HPβCD-CLOT-IC and PUR3/HPβCD-CLOT-IC, FTIR was applied. Since all the PURs prepared herein are cross-linked with HPβCD as chain extenders, similar spectra were found for all synthesized PURs. FTIR of PUR2/HPβCD and PUR3/HPβCD from the previous study [15,16] were used for the evaluation of the new results.

**Figure 3:**
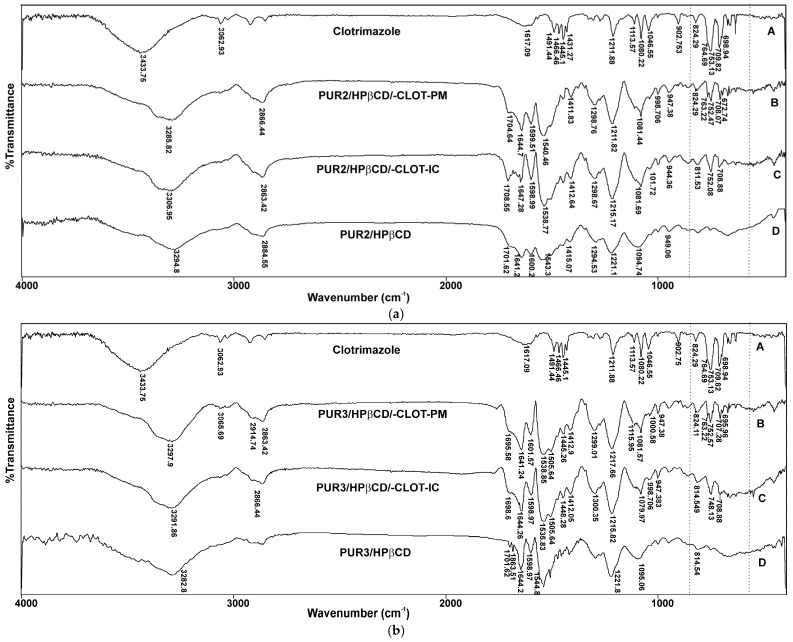
FTIR of pure clotrimazole, physical mixtures (PM), and inclusion complexes (IC) of clotrimazole with cross-linked polyurethanes, (**a**) PUR2/HPβCD and (**b**) PUR3/HPβCD.

Characteristic FTIR absorption peaks of pure clotrimazole (Figure 3a) such as aromatic C–H stretch (3062.9 cm^−1^), aromatic C=C stretch (1617.1 and 1491.4 cm^−1^), C=N stretch (1466.4 cm^−1^), aromatic C–H bending (753.1 cm^−1^), C–Cl cyclic (824.2 cm^−1^), and vibration band of C–H (1211.8 cm^−1^) were present in the spectrum of pure powder CLOT and consistent with the literature [27].

Moreover, specific PEG bands can also be observed around 1410 cm^−1^ (O–H), 1300 cm^−1^ (O–H), 1200 cm^−1^ (C–H), and 1080 cm^−1^ (C–O).

Spectra C in Figure 3a,b, shows the representative FTIR observed for the HPβCD-modified polyurethane polymers (PUR2/HPβCD and PUR3/HPβCD) and confirms the identity of the synthesized polymer. The most noteworthy feature in the FTIR is the disappearance of the isocyanato group around 2270 cm^−1^ and the appearance of signals corresponding to amide vibrational bands (3294.8, 1701.6 and 1600 cm^−1^ corresponding to N–H, C=O, and C–N, respectively). These results supported that the reaction was successful in the formation of a urethane linkage between HPβCD and the isocyanate cross-linking agent [28].

Unlike the FTIR of the inclusion complex (spectra C in Figure 3a,b), significant changes in the spectrum of the physical mixture (Figure 3b) were noticed by comparing with the spectra of the pure substances. It can be clearly observed that the FTIR for the physical mixture is almost identical to that of the cross-linked polyurethane with cyclodextrins and clotrimazole [16]. The positions of characteristic peaks of clotrimazole (e.g., peak from C–Cl stretching vibration around 824 cm^−1^) were not altered after their successful entrapment in the physical mixture, suggesting the absence of any specific chemical interactions between the drug and other components of the formulation.

On the other hand, in the FTIR of the inclusion complex, clotrimazole’s aromatic region peaks at around 600 to 850 cm^−1^ have disappeared, which suggested that these groups of the drug were included in the cross-linked polyurethane networks based on HPβCD [16,29]. The hydrophobic part of HPβCD showed affinity for the hydrophobic aromatic rings of CLOT and formed a complex due to the possible hydrogen bonds between the secondary hydroxyl group of HPβCD and the imidazole nitrogen, as well as van der Waals forces of attraction [30].

### 3.3. Wide-Angle X-Ray Diffraction (XRD)

**Figure 4:**
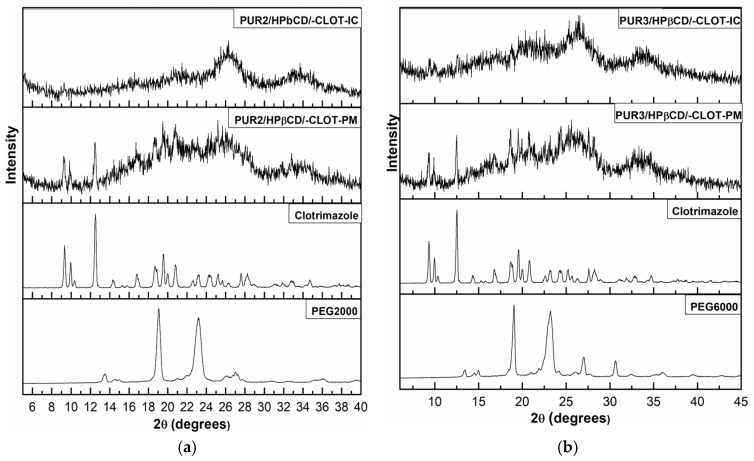
X-ray diffraction patterns of pure clotrimazole, physical mixtures (PUR2/HPβCD/-CLOT-PM and PUR3/HPβCD/-CLOT-PM), polyol PEG 2000, polyol PEG 6000, and inclusion complexes (IC) of clotrimazole with HPβCD-modified polyurethane: (**a**) PUR2/HPβCD/-CLOT-IC and (**b**) PUR3/HP*β*CD/-CLOT-IC.

X-ray powder diffractometry patterns of the clotrimazole, physical mixtures, and inclusion complexes clotrimazole: HPβCD-modified polyurethane networks are shown in Figure 4. The diffraction spectrum of pure clotrimazole showed that the drug was crystalline in nature as demonstrated by numerous peaks at 9.3°, 10.0°, 12.5°, 18.7°, 19.5°, 20.7°, 23.1°, and 25.2°. X-ray diffraction patterns of pure PUR2/HPβCD, PUR3/HPβCD, and polyol PEG 2000 from the previous study [15,16] were used for comparison with the new results.

As seen in Figure 4, diffractograms of the physical mixtures of samples PUR2/HPβCD/-CLOT-PM and PUR3/HPβCD/-CLOT-PM with clotrimazole showed specific peaks at 9.3°, 10.0°, 12.5°, 18.7°, 19.5°, 20.7°, and 23.1°, which corresponded to a pure drug CLOT [27,29]. Because the diffraction maxima presence in the physical mixture was identical with those of the pure drug, it can be suggested that there was no interaction between the constituents [31,32].

In contrast to the physical mixture, diffraction patterns of the inclusion complexes of samples PUR2/HPβCD/-CLOT-IC and PUR3/HPβCD/-CLOT-IC with CLOT (prepared by the kneading method) indicated a lack of a diffraction maximum derived from CLOT, which could prove that this compound did not occur in the crystalline form [27].

Complexes were characterized by diffraction maxima of very low intensity, and the intensity of diffraction maxima was close to the intensity of the diffraction maximum of HPβCD or the used polyols. These results indicated that CLOT was no longer present as a crystalline material, and its HPβCD–modified polyurethane solid complexes exist in the amorphous state. The broadening of some CLOT crystalline peaks and disappearance of others confirmed the stronger drug amorphization and entrapment into the cavity of cyclodextrins due to the combined action of HPβCD and the cross-linked polymers [27,33]. The formation of an amorphous state proved that CLOT was dispersed in a molecular state within an inclusion complex polyurethane based on HPβCD and cross-linked chains of modified polymer networks. Therefore, the CLOT inclusion complexes with cross-linked polyurethanes modified with HPβCD (samples PUR2/HPβCD/-CLOT-IC and PUR3/HPβCD/-CLOT-IC) were amorphous.

### 3.4. Thermogravimetric Analysis (TGA)

**Figure 5:**
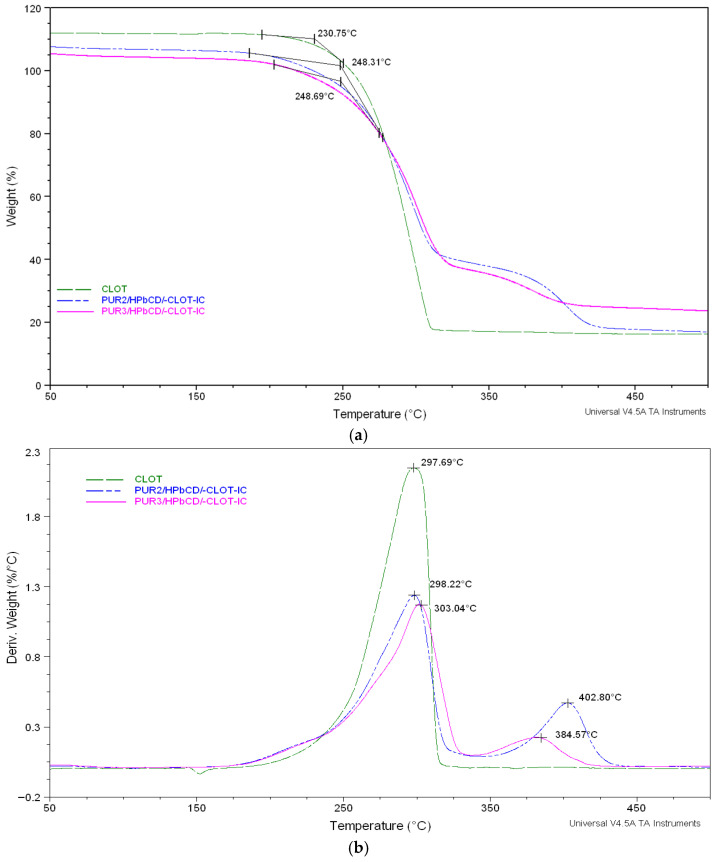
TG (**a**) and DTG (**b**) thermograms of the pure clotrimazole and inclusion complexes (IC) of clotrimazole: HPβCD-modified polyurethane networks (PUR2/HPβCD-CLOT-IC and PUR3/HPβCD-CLOT-IC).

**Figure 6:**
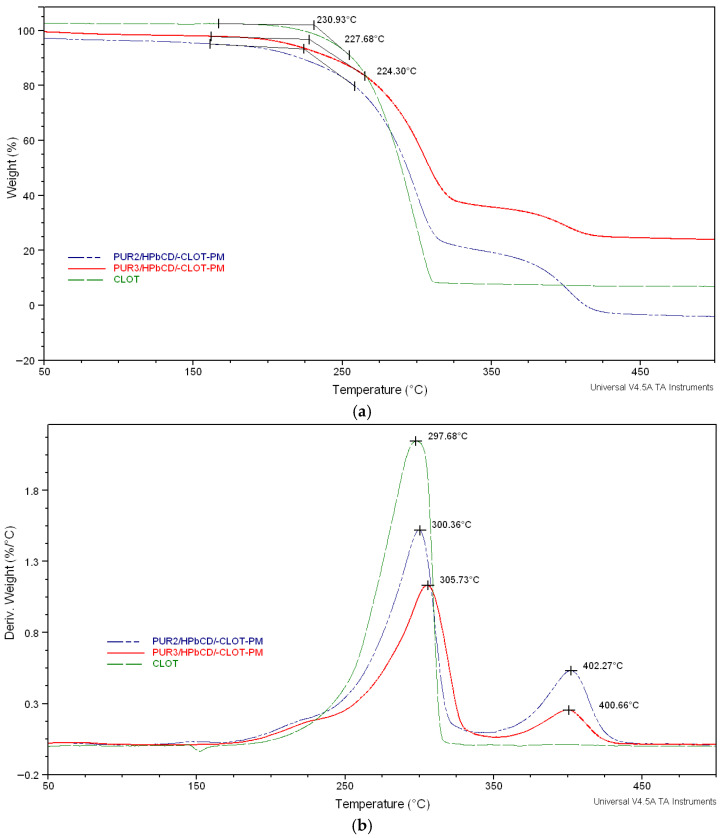
TGA thermograms (**a**) and their derivatives (**b**) of the pure clotrimazole and physical mixtures (PM) of clotrimazole and HPβCD-modified polyurethane networks (PUR2/HPβCD-CLOT-PM and PUR3/HPβCD-CLOT-PM).

The effect of polyol structure (PEG 2000 or PEG 6000) on the stability of inclusion complexes of polyurethane series based on HPβCD and clotrimazole in a nitrogen atmosphere is illustrated in Figure 5 and Figure 6.

As shown in these figures, all TGA curves displayed a slower, two-step degradation mechanism under a nitrogen atmosphere. From the TGA curves, it was found that degradation was comparatively slow in the cross-linked polyurethane polymers, indicating that they were reasonably stable up to their melting points. The initial degradation depends on the hard segments’ structure, and the urethane linkage degraded first at 220–330 °C; consequently, the second step of degradation was associated with the soft segment degradation, which started at above 300 °C [5,27].

The TGA thermogram of the drug clotrimazole showed a major degradation temperature at 230.75 °C/230.93 °C (Figure 5a and Figure 6a).

The series of inclusion complexes between cross-linked polyurethane networks and clotrimazole based on HPβCD displayed similar degradation profiles, suggesting that changes in the soft segment molecular weight (PEG 2000, PEG 6000) and NCO/OH ratio caused a slight increase in thermal stability (decomposition temperatures rises from 244.74 and 248.31 to 248.69 °C) while increasing the NCO/OH molar ratio (i.e., isocyanate content) [12,13,27]. Nevertheless, the first weight loss maximum of PUR3/HPβCD/-CLOT-IC (303.04 °C) was higher than that of PUR2/HPβCD/-CLOT-IC (296.22 °C).

In contrast, the lower stability for physical mixture may be attributed to the lower extent of interurethane hydrogen bonding arising from the incomplete phase separation between the soft or hard segments and clotrimazole, as well as an inferior mutual stabilization effect in the CLOT-loaded cross-linked polyurethane networks. The TG curves (Figure 6a,b) for a series of physical mixtures showed a similar trend, with decomposition temperatures rising from 224.30 to 227.68 °C for samples PUR2/HPβCD/-CLOT-PM and PUR3/HPβCD/-CLOT-PM, respectively [27].

The TGA thermogram results indicated a slightly increased thermal stability of the clotrimazole loading in inclusion complexes cross-linked polyurethane networks (PUR2/HPβCD/-CLOT-IC and PUR3/HPβCD/-CLOT-IC) [27].

The series inclusion complexes of clotrimazole and HPβCD-modified polyurethane networks displayed similar degradation profiles, suggesting that changes in soft segment molecular weight (PEG 2000, PEG 6000) and NCO/OH ratio caused a slight increase in thermal stability. It had been expected that inclusion complexes would have a longer drug release with higher degrees of cross-linking.

Generally, an increase in isocyanate content (increase in the NCO/OH molar ratio from 4.3 to 6.3) increased the cross-linking and thermal stability for physical mixtures and inclusion complexes of PURs based on HPβCD and clotrimazole [2,26,31,32,33].

### 3.5. DSC Analysis

**Figure 7:**
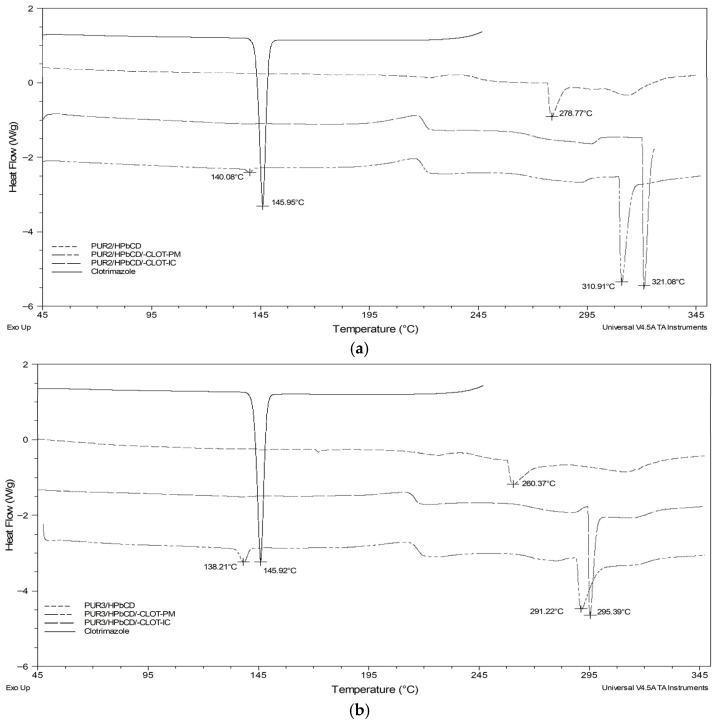
DSC thermograms of the pure clotrimazole (CLOT) and: (**a**) HPβCD-modified polyurethane network (PUR2/HPβCD), physical mixture of CLOT and PUR2/HPβCD (PUR2/HPβCD-CLOT-PM), and inclusion complex of CLOT and PUR2/HPβCD (PUR2/HPβCD-CLOT-IC); (**b**) HPβCD-modified polyurethane network (PUR3/HPβCD), physical mixture of clotrimazole and PUR3/HPβCD (PUR3/HPβCD-CLOT-PM), and inclusion complexes of CLOT and PUR3/HPβCD (PUR3/HPβCD-CLOT-IC).

The thermal transition properties of the physical mixtures (PUR2/HPβCD-CLOT-PM and PUR3/HPβCD-CLOT-PM) and the inclusion complexes of clotrimazole with HPβCD-modified polyurethane networks (PUR2/HPβCD-CLOT-IC and PUR3/HPβCD-CLOT-IC) were investigated by means of differential scanning calorimetry (DSC) and compared with both each other and the thermograms of the pure clotrimazole and PUR2/HPβCD or PUR3/HPβCD (Figure 7a,b).

The DSC curve of clotrimazole showed one characteristic sharp endothermic peak at around 145.90 °C, indicating the melting point of the drug.

The endothermic peaks of the soft segment melting were also observed in cross-linked polyurethanes PUR2/HPβCD and PUR3/HPβCD at 278.77 °C and 260.37 °C, respectively, as shown in Figure 7a,b [25].

The melting point for the inclusion complexes PUR2/HPβCD/-CLOT-IC (321.08 °C), and PUR3/HPβCD/-CLOT-IC (295.39 °C) was higher than their physical mixtures of PUR2/HPβCD/-CLOT-PM (310.91 °C) and PUR3/HPβCD/-CLOT-PM (291.22 °C), which was attributed to either an increase in the size of the crystallites or an increase in the complete crystalline phase [34,35].

The characteristic endothermic peak of the clotrimazole did not appear in the thermogram of the drug-loaded inclusion complexes PUR2/HPβCD-CLOT-IC and PUR3/HPβCD-CLOT-IC. This suggested the drug had been molecularly dispersed and entrapped within amorphous domains of the cross-linked polyurethane networks and within the cavities of HPβCD [2,26,32].

The DSC curves of the physical mixtures PUR2/HPβCD-CLOT-PM and PUR3/HPβCD-CLOT-PM were the superpositions of the curves of the two individual components, containing peaks representative of both PUR2/HPβCD or PUR3/HPβCD and CLOT alone (Figure 7a,b). There was no signal indicative of a thermally induced interaction between the two components.

The endothermic peak of CLOT decreased in the physical mixtures PUR2/HPβCD-CLOT-PM and PUR3/HPβCD-CLOT-PM, but the characteristic peak of CLOT could be recognized [27,36].

### 3.6. SEM Analysis

**Figure 8:**
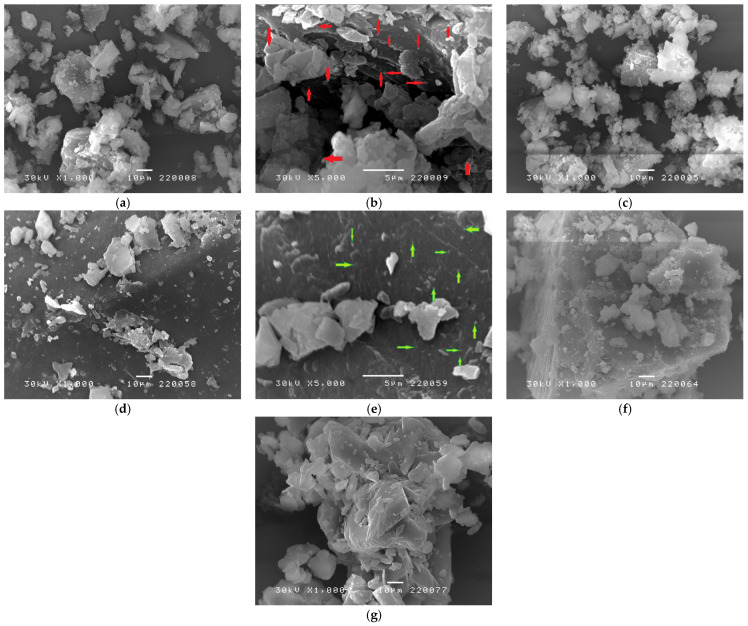
SEM micrographs of analyzed samples: (**a**) PUR2/HPβCD-CLOT-IC (magnification 1000×, barr 10 μm); (**b**) PUR2/HPβCD-CLOT-IC (magnification 5000×, bar 5 μm); (**c**) PUR2/HPβCD-CLOT-PM (magnification 1000×, bar 10 μm); (**d**) PUR3/HPβCD-CLOT-IC (magnification 1000×, bar 10 μm); (**e**) PUR3/HPβCD-CLOT-IC (magnification 5000×, bar 5 μm); (**f**) PUR3/HPβCD-CLOT-PM (magnification 1000×, bar 10 μm); and (**g**) CLOT (magnification 1000×, bar 10 μm).

The surface morphology of the host–guest inclusion complexes of clotrimazole and HPβCD-modified polyurethane networks (PUR2/HPβCD-CLOT-IC and PUR3/HPβCD-CLOT-IC) and their corresponding physical mixtures (PUR2/HPβCD/-CLOT-PM and PUR3/HPβCD/-CLOT-PM) are presented in SEM micrographs (Figure 8a–f), as well as comparisons to each other and with the morphology of pure clotrimazole (Figure 8g).

Morphological analysis of the SEM micrograph of clotrimazole indicated particles with irregular, planar structures and broader size distribution, indicating its amorphous-crystalline and highly lipophilic nature (Figure 8g) [32].

The particle shape of dry powder samples of the inclusion complexes between clotrimazole and HPβCD-modified polyurethanes based on polyols PEG 2000 or PEG 6000 (PUR2/HPβCD-CLOT-IC and PUR3/HPβCD-CLOT-IC, Figure 8a,b and Figure 8d,e, respectively) indicated fragmented synthesized structures that differed from micrographs of pure CLOT or the corresponding physical mixtures (PUR2/HPβCD-CLOT-PM and PUR3/HPβCD-CLOT-PM, Figure 8c and Figure 8f, respectively), as well as the corresponding pure HPβCD-modified polyurethanes (before CLOT inclusion) described earlier [25].

Irregular, plate-shaped structures, in the form of smaller or larger hard PUR segment agglomerates, and elliptical/spherical-shaped particles (originating from the hard and soft HPβCD-modified polyurethane segment lengths with included CLOT) were present without phase segregation [37,38,39]. The particle size (according to the bar scale) showed a wide size distribution, where the smallest particles (about 0.1 µm), small particles and smaller agglomerates (about 1–5 µm), and larger agglomerates (between 5 and 15 µm) indicated the presence of heterogeneous networks with local differences in their densities (Figure 8b,e with magnification 5000×). Their surfaces indicated a rough, irregular texture with cracks and stepped fracture planes but no clearly defined pores, which could increase the specific surface area and facilitate the penetration of the medium in the clotrimazole release phase. The PUR3/HPβCD-CLOT-IC samples had a slightly rougher surface compared to the PUR2/HPβCD-CLOT-IC samples with a more uniform surface, which could be attributed to the increased cross-linking density and the longer length of the polyol chains/soft segments. SEM micrographs of the inclusion complexes showed the altered topology and indirectly confirmed that CLOT was not present as a separate phase due to their inclusion into the HPβCD cavities; in addition, CLOT was trapped/embedded by physical bonds in the hollows between the cross-linked PUR chains of PUR2/HPβCD-CLOT-IC and PUR3/HPβCD-CLOT-IC [40,41,42]. SEM analysis indicated that the formed inclusion complexes clotrimazole: HPβCD-modified polyurethane networks were not classically porous, but rather a diffusional active and fragmented with interparticle spaces.

### 3.7. In Vitro Clotrimazole Release Study

**Figure 9:**
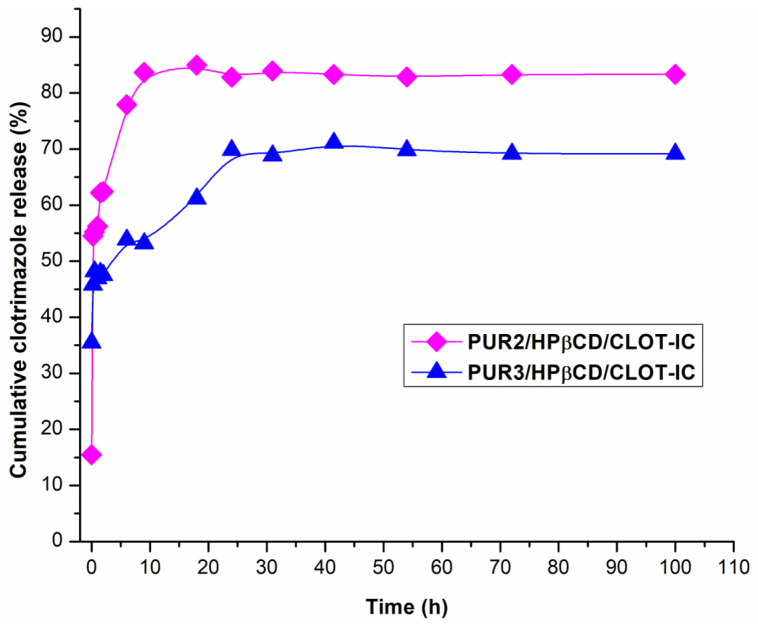
In vitro cumulative clotrimazole release profiles from inclusion complexes clotrimazole: HPβCD-modified polyurethane networks PUR2/HPβCD-CLOT-IC (pink diamonds) and PUR3/HPβCD-CLOT-IC (blue triangles), performed in pH 6.8 in dependence on time during 100 h.

To explore the potential use of HPβCD-modified cross-linked polyurethane networks as controlled clotrimazole delivery systems, inclusion complexes of clotrimazole: cross-linked HPβCD-modified polyurethane networks were evaluated. In vitro cumulative release profiles of clotrimazole from inclusion complexes PUR2/HPβCD-CLOT-IC and PUR3/HPβCD-CLOT-IC in a slightly alkaline fluid at pH 6.8 and 37 °C in dependence on time were monitored for 4 days, and the obtained results are illustrated in Figure 9.

The content of clotrimazole released during an average time of 100 h in simulated physiological conditions from obtained samples of inclusion complexes showed higher values of released drug from PUR2/HPβCD-CLOT-IC (83.32%, i.e., 6.92 mg_clot_/g_ic_ of the loaded drug amount, which was 8.3 mg_clot_/g_ic_) in relation to PUR3/HPβCD-CLOT-IC (69.16%, i.e., 5.74 mg_clot_/g_ic_) (Figure 9). Under the defined conditions, an initial burst release of about half of the clotrimazole mass was released within the first 15 min (54.48% and 45.76% from PUR2/HPβCD-CLOT-IC and PUR3/HPβCD-CLOT-IC samples, respectively). During the following periods, the remaining amount of CLOT released more slowly, i.e., from 62.41% and 47.51% after 2 h to 82.82% and 69.80% after the first 24 h from PUR2/HPβCD-CLOT-IC and PUR3/HPβCD-CLOT-IC, respectively (Figure 9). During the monitored period (from the second to the fourth days), CLOT was released continuously. These findings indicated that CLOT was likely to first be released rapidly from the intermolecular space of the polyurethane network, and then more slowly from the cavities of the HPβCD. Analysis of these results suggested that host–guest inclusion complexes obtained using PEG 2000 had a lower density network and a larger internal free volume, which allowed a greater amount of CLOT to be released compared to the PUR3/HPβCD-CLOT-IC sample over the same time. The greatest influence on this behavior was the length of the polymer chains of the soft segments, increasing the molar ratio of NCO/OH from 4.3 to 6.3, which led to an increase in the cross-linking density. The soft segments originating from PEG 2000 had a shorter length, which enabled less elasticity of the polymer chains in the amorphous zones and thus, a lower possibility of retention CLOT within the polymer matrix. In contrast, PEG 6000 influenced the formation of additional interwoven zones involving more inter- and intramolecular interactions within the hard and the soft PUR segments. For this reason, a slightly smaller amount of CLOT was released from the PEG 6000-based sample, which has a higher network density and smaller internal free volume between the nodes of cross-linked chains. As a result, a slightly larger amount of included CLOT remained trapped by the physical bonds within the formed network and allowed for a slow, prolonged release of the remaining drug for more than 4 days. The phenomenon where CLOT was released more slowly from this sample (PUR3/HPβCD-CLOT-IC) compared to the PUR2/HPβCD-CLOT-IC may indicate stronger intermolecular interactions with the side groups of the modified polyurethane network, so they probably needed more time to pass through the more voluminous soft and flexible segments, representing the transport channels that can regulate drug release. Such incorporations were also favored by the relatively small size of the CLOT molecule (344.84 g/mol molar mass) with characteristic functional groups available for intermolecular interactions.

FTIR analysis confirmed that some absorption bands originating from CLOT in the spectrum of the host–guest complexes were covered due to their inclusion in the cavities of HPβCD, trapping them within the cross-linked polyurethane network (loss of peaks from the aromatic region of clotrimazole from 600 to 800 cm^−1^) [28] whereby hydrogen bonds and van der Waals forces of attraction were formed [29].

Since the same system considered in this study was not found in the available literature, some of the similar drug delivery systems with their drug loading efficiency, amount of released drug, release time, and drug release kinetics are presented in Table 1.

The rate of drug release from the interior of HPβCD, as well as from the polymer network, was dependent on the intermolecular bonds between the polymer and the drug, which has also been shown in studies by other authors. For example, a study by de Araújo and colleagues showed that cross-linked polyurethane networks based on HPβCD and polyethylene glycols PEG 1500 achieved 80% nifedipine (NIF) release in an average dissolution time of 9.42 h from the sample PUR/TDI/HPβCD/PEG1500-NIF at a slightly acidic fluid (pH 6.8) [5]. They found that inclusion complexes between cross-linked polyurethane networks and nifedipine exhibited a significantly slower release rate than pure nifedipine. A particularly surprising fact when comparing the drug release times was that the release time of CLOT from a similar carrier in this study with a slightly longer chain length (of PEG 2000), was 10.6 times longer than that of NIF from the de Araújo study. Nifedipine has a molar mass of approximately 346.34 g/mol (with pyridine ring and nitrophenyl group), very similar to that of clotrimazole of 344.8 g/mol. The significant differences in the results were probably influenced by certain differences between the reactants and the different times of the second phase of the polymerization process. de Araújo used the monomer tolylene diisocyanate, the solvent dimethylformamide, and the catalyst stannous octanoate, while in this work isophorone diisocyanate, dimethyl formamide, and dibutyltin dilaurate were used. These results indicated that the drug inclusion capacity and its release were greatly influenced by the length and conformation of the soft segments and the incorporation of the drug into the cross-linked polyurethane networks, as well as the formation of a host–guest inclusion complex (Figure 2). It has been observed that the inclusion capacity for potential use as a controlled release carrier also depended on the nature of the drug (molecular weight, functional groups).

The hydrophilic properties of HPβCD could provide numerous hydrogen bond formations with water molecules and consequently faster drug release rates [5,52]. The solubility of polyethylene glycol increased as the molecular weight decreased, probably due to a more hydrophilic environment, which enhanced the drug release rate compared with the PEG sample with a higher molecular weight [33].

It was found that the drug release rate was inversely proportional to the molar ratio of NCO/OH, and directly dependent on the ratio of polyol/cross-linkers and polyol/chain extenders [2,33]. These findings indicated that designing the polyurethane matrix by varying the polymer chain length and the NCO/OH ratio can affect the drug release rate (in this case clotrimazole), in addition to improving its aqueous solubility and bioavailability, which may have important clinical implications and a more effective pharmacological response.

As stated, the loading efficiency of CLOT in the present study was ~16.6%, while the release time was monitored for 4 days. A comparative review of the loading efficiency (Table 1) varied depending on the type of polymer and its structure, preparation method, and drug type (molecular mass, functional groups), suggesting the importance of careful selection for the appropriate preparation method to optimize the encapsulation of various bioactive compounds. For example, the lowest values of 5% felodipine with almost insignificant differences among the three systems of polyurethanes/poly(ε-caprolactone) were lactic acid, glycolic acid, or dimethylol propionic acid, which released during 24 h 98.52%, 80.29%, and 54.69% of the drug, respectively [33]. The highest loading efficiency values were achieved for praziquantel into poly(methyl methacrylate) nano- and microparticles (100%) as well as poly(methyl methacrylate)–*co*–(2-(dimethylamino)ethyl methacrylate) and poly(methyl methacrylate)–*co*–(2-(diethylamino)ethyl methacrylate) microparticles (99%) [50]. Similarly, eugenol into β-CDs complexes obtained by kneading reached loading efficiency values of 99.4% [53], and clotrimazole into solid dispersions of polyvinyl pyrrolidone and PEG 4000 with βCD reached 99% [51]. The slowest release time was achieved for meropenem from 1,6-hexamethylenediisocyanate at 10% for 5 days, although the loading efficiency was 82 ± 4% [49] (Table 1). Most of the other monitored systems released their loaded drugs during 120 min [51], 180 min [52], 4 h [54], 12 h [5], and 24 h [32,55,56,57] periods.

### 3.8. Mathematical Modeling of Clotrimazole Release

**Figure 10:**
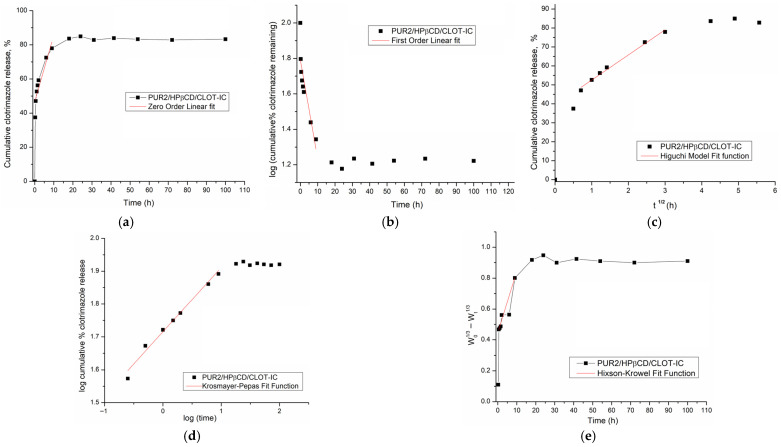
The release kinetics model fitting curves of clotrimazole release from HPβCD-modified polyurethane inclusion complexes PUR2/HPβCD-CLOT-IC: (**a**) zero-order release kinetics; (**b**) first-order kinetics model; (**c**) Higuchi model; and (**d**) Korsmeyer–Peppas model and (**e**) Hixson–Crowell model.

**Figure 11:**
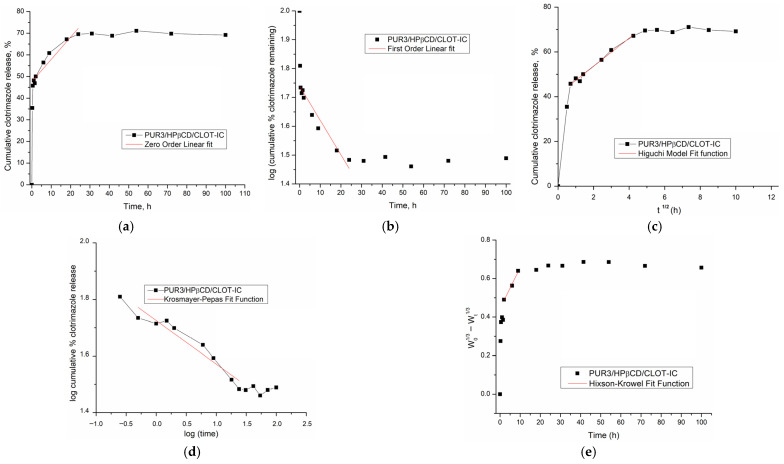
The release kinetics model fitting curves of clotrimazole release from HPβCD-modified polyurethane inclusion complexes PUR3/HPβCD-CLOT-IC: (**a**) zero-order release kinetics; (**b**) first-order kinetics model; (**c**) Higuchi model; (**d**) Korsmeyer–Peppas model and (**e**) Hixson–Crowell model.

In order to compare the release profiles of the total amount of clotrimazole released over specified intervals of time from cross-linked HPβCD-modified polyurethane inclusion complexes (PUR2/HPβCD-CLOT-IC and PUR3/HPβCD-CLOT-IC), as well as to understand its release kinetics, zero-order, first-order, Higuchi, Korsmayer–Peppas, and Hickson–Crowell mathematical models were applied [53,54,55,56]. The obtained kinetic parameters are summarized in Table 2 and Figure 10 and Figure 11.

Mathematical models have a significant role in understanding the mechanism and kinetics of drug release from the dosage form. For the best model selection, the correlation coefficient (*R*^2^) and graphical adjustment were taken into account [52].

Zero-order and first-order models can be applied for the 50% of the drug release based on the related data [53]. The graphical representation of the *log* of cumulative drug release *versus* time detailed that the release of CLOT from the HPβCD-modified polyurethane networks did not follow the principles of the zero- and first-order release kinetics (*R*_adj_^2^ are 0.7949 and 0.7326 for PUR2/HPβCD-CLOT-IC and 0.9202 and 0.888 for PUR3/HPβCD-CLOT-IC, respectively).

Higuchi and Korsmeyer–Peppas models can generate a fit function up to 60% of drug release, which was the end of the release data [57].

The Higuchi model indicated that CLOT release from both HPβCD-modified polyurethane inclusion complexes was governed by Fickian diffusion. This indicated that CLOT was uniformly dispersed within a stable matrix that acted as a diffusion reservoir, with no significant erosion occurring. Furthermore, CLOT release followed a square-root-of-time dependence characteristic of a diffusion-controlled process.

The Korsmeyer–Peppas model describes drug release from a polymer system, depending on the type of dissolution. The value of the diffusion exponent, *n*, indicated the drug release mechanism (*n* ≤ 0.45—Fickian diffusion; 0.45 < *n* < 0.89—anomalous behavior, non-Fick diffusion; *n* ≥ 0.89—zero-order release). The diffusion exponents *n* of 0.1956 and 0.1537, calculated for PUR2/HPβCD-CLOT-IC and PUR3/HPβCD-CLOT-IC, respectively, showed that the release mechanism of clotrimazole was based on diffusion from the polymer matrix, and the transport process of CLOT was controlled by the Fickian diffusion mechanism as a slower process—the so-called Case I. This parameter was a measure of how fast CLOT moved through an HPβCD-modified polyurethane network and confirmed the slower drug release from the PUR3/HPβCD-CLOT-IC with PEG 6000. Values of *n* around 0.32 or lower (0.1537–0.1956) were specific for systems where molecules were “caged” in dense networks [58]. In partially interwoven polyurethane amorphous segments, the chains were constrained by surrounding crystal segments (Figure 2), which was the cause of restricted diffusion.

The Hickson–Crowell model describes the drug release from a system in which the surface area and particle diameter decreased over time due to progressive matrix dissolution. According to calculated results (*R*_adj_^2^ are 0.9715 and 0.9808 for PUR2/HPβCD-CLOT-IC and PUR3/HPβCD-CLOT-IC, respectively), the Hickson–Crowell model was not dominant, indicating that erosion or surface degradation was not the prevailing mechanism, and rather, diffusion governed the process [10].

A summary of some of the literature data based on the kinetic mechanism in different drug delivery systems is presented in Table 1. Linear fitting of the release data according to the Korsmeyer–Peppas model, as reported in the study by Diaconu et al. on levofloxacin release from polyurethane–cyclodextrin–oligocaprolactone-based hydrogels, revealed diffusion exponent (*n*) values in the range of 1.35–1.48, indicating the Super Case II transport of drug release mechanism [16]. The levofloxacine amount loaded in the hydrogels was 8.33–20.77 mg. Starch-incorporated beads of chitosan-*graft*-polyethylene glycol showed slower release rates of clotrimazole than either chitosan or chitosan-*graft*-polyethylene glycol matrices. The release of CLOT from these beads followed a less Fickian diffusional release mechanism and zero-order kinetics [1]. The in vitro release of clotrimazole from its polyvinylpyrrolidone solid dispersions and ternary clotrimazole-β-cyclodextrin inclusion complexes incorporated suppositories were noticeably improved [36]. The nifedipine release, according to the de Araújo study, was explained by a biexponential release model (the first stage is a rapid/burst effect, and the second stage is a slow/controlled release) [5].

## 4. Conclusions

Inclusion complexes of clotrimazole and HPβCD-modified polyurethane network based on polyols PEG 2000 and PEG 6000 were successfully prepared. New, covalently cross-linked structures were formed using urethane bonds. Also, inclusion/incorporation of the hydrophobic drug clotrimazole with physical bonds was achieved, which was confirmed by FTIR analysis and comparison with FTIR results of corresponding physical mixtures and CLOT. XRD diffractograms of physical mixtures showed characteristic peaks corresponding to pure CLOT, but they disappeared in inclusion complexes of clotrimazole and HPβCD-modified polyurethane, mainly due to the physical cross-linking process. SEM analysis indicated that the inclusion complexes clotrimazole: HPβCD-modified polyurethane networks were fragmented with interparticle spaces and a wide size distribution from the smallest particles (about 0.1 µm) to small particles and agglomerates. The obtained in vitro results showed slow/prolonged clotrimazole release from HPβCD-modified polyurethane networks with potential in vivo application. The special significance of this work was in the analysis of the influence of the molar ratio NCO/OH and chain lengths of soft segments, which affected the structural characteristics of the polymer networks, and in particular, conditioned the transfer of drug molecules through the carrier and the surprisingly slow release of CLOT. The Higuchi model best described drug release kinetics, implying that CLOT release from the HPβCD-modified polyurethane networks was a diffusion-controlled process. These experimental results can provide the basis for further analysis and development of potential formulations for the preparation of numerous conventional dosage forms (ointments, creams, vaginal suppositories, and solutions) intended for topical or vaginal administration with prolonged delivery of CLOT for 100 h (4 days).

## 5. Patents

Patent Application RS-P-2026-0310. Cakić, S.; Nikolić, L.; Nikolić, V.; Ristić, I.; Nikolić, N.; Ilić-Stojanović, S. Process for obtaining inclusion complexes of cyclodextrins modified with polyurethane networks for sustained release of clotrimazole, the Intellectual Property Office of the Republic of Serbia.

## Figures and Tables

**Table 1:** Summary of different drug delivery systems, their drug loading efficiency, amount of released drug, %, release time, and drug release kinetics.

Carrier	Drug	Loading Efficiency, %	Drug Release, %, and Release Time	Drug Release Kinetic	Refs
Polyurethanes/poly(ε-caprolactone) with lactic acid/glycolic acid/dimethylol-propionic acid	Felodipine	5.0562, 5.0532, and 5.0548	98.52%, 80.29%, 54.69% during 24 h	Fickian diffusion	[33]
2-hydroxypropyl-β-cyclodextrin/ 1,6-hexamethylenediisocyanate	Meropenem	82 ± 4%	10% during 5 days	–	[43]
Poly(methyl methacrylate) and (2-(diethylamino)ethyl methacrylate) or (2-(dimethylamino)ethyl methacrylate)/nano- and microparticles	Praziquantel	100 (nano) 96 and 99 (microparticles)	92.17 ± 5.3% during 180 min	Anomalous (diffusion and relaxation)	[44]
PUR/TDI/βCD/PEG4000-NIF	Nifedipine	-	80% during 12 h	Biexponential	[5]
PUR2/HPβCD-CLOT-IC, PUR3/HPβCD-CLOT-IC	Clotrimazole	16.60–16.64	83.32%, 69.16% (pH 6.8) during 4 days	Higuchi	
Poly(ethylene oxide)-block-poly(ε-caprolactone) copolymer micelles	Clotrimazole	71.81, 87.95 and 80.12	78 ± 1.43% during 120 h (5 days)	-	[45]
Carboxylate cross-linked β-cyclodextrin nanosponges	Rosuvastatin calcium	88.76	~80% (pH 1.2) ~100% (pH 6.8) during 4 h	-	[46]
Poly(lactic-*co*-glycolide), poly(vinyl alcohol) and poly(L-lysine)	Curcumin	49.56 ± 4.52 89.53 ± 3.26	64.37–78.45%, 86.73–98.92, during 24 h	Huguchi	[47]
β-CDs complexes obtained by kneading, co-precipitation, lyophilization, co-precipitation and lyophilization	Eucalyptol, Eugenol, Clove essential oil	48.68–95.62 55.01–99.38 60.48–99.40	-	-	[48]
Eudragit^®^ RS100 (poly(methyl methacrylate-methacrylic acid) nanoparticle	Clotrimazole	-	66% and 58% during 24 h	Anomalous transport	[49]
Carbopol 934P/sodium alginate sodium carboxymethyl cellulose	Clotrimazole	-	99% during 24 h	Zero-order	[50]
Solid dispersions of polyvinyl pyrrolidones, PEG 4000 with βCD	Clotrimazole	96 and 99	27.9%, 33.21% and 33.2% during 120 min	Zero-order	[51]

**Table 2:** Kinetic parameters and statistics of kinetic models for clotrimazole release from cross-linked HPβCD-modified polyurethane inclusion complexes PUR2/HPβCD-CLOT-IC and PUR3/HPβCD-CLOT-IC in the simulated physiological fluid at pH 6.8 and 37 °C.

Kinetic Model Name and Equation	PUR2/HPβCD-CLOT-IC			PUR3/HPβCD-CLOT-IC		
	*R*_adj_^2^	Slope *	Intercept	*R*_adj_^2^	Slope *	Intercept
Zero-order model F = *k*_0_·*t*	0.7949	3.8812	46.784	0.9202	1.0269	47.6463
First-order model F = 100·[1 − *e*^−*k*1·*t*^]	0.7326	−0.0556	1.7902	0.8880	−0.0119	1.7395
Higuchi model F = *k*_H_·*t*^1/2^	0.9890	13.2399	39.3214	0.9840	6.2747	41.0263
Korsmeyer–Peppas model F = *k*_KP_·*t*^n^	0.9762	0.1956	1.7155	0.9116	0.1537	1.7254
Hixson–Crowell model F = 100(1 − (1 − *k*_HC_·*t*)^3^)	0.9715	0.0396	0.4476	0.9808	0.0212	0.4444

* F is the cumulative clotrimazole release; slope are the release constants depending on the model: *k*_0_, −*k*_1_, *k*_H_, *n = k*_KP_, −*k*_HC_; and *R*_adj_^2^ is the adjusted coefficient of determination.

## Data Availability

The original contributions presented in this study are included in the article. Further inquiries can be directed to the corresponding author.

## Abbreviations

The following abbreviations are used in this manuscript:

HPβCD	Hydroxypropyl-*β*-Cyclodextrin
CDs	Cyclodextrins
PURs	Polyurethanes
CLOT	Clotrimazole
PEG	Polyethene Glycol
IPDI	Isophorone Diisocyanate
PUR2/HPβCD	Cross-linked HPβCD-modified Polyurethane based on PEG 2000
PUR3/HPβCD	Cross-linked HPβCD-modified Polyurethane based on PEG 6000
DMF	Dimethyl Formamide
DBTDL	Dibutyltin Dilaurate
PUR2/HPβCD-CLOT-IC	Inclusion Complexes of Clotrimazole with PUR2/HPβCD
PUR3/HPβCD-CLOT-IC	Inclusion Complexes of Clotrimazole with PUR3/HPβCD
PUR2/HPβCD-CLOT-PM	Physical Mixture of Clotrimazole and PUR2/HPβCD
PUR3/HPβCD-CLOT-PM	Physical Mixture of Clotrimazole and PUR3/HPβCD
FTIR	Fourier Transform Infrared Spectroscopy
DTA	Differential Thermal Analysis
TGA	Thermogravimetric Analysis
DSC	Differential Scanning Calorimetry
XRD	Wide-angle X–ray Diffraction
SEM	Scanning Electron Microscopy
HPLC	Reverse-Phase High-Performance Liquid Chromatography

## References

[1] Akakuru O.U., Louis H., Uwaoma R., Elemike E.E., Akakuru O.C. (2019). Novel highly-swellable and pH-responsive slow release formulations of clotrimazole with chitosan-g-PEG/starch microparticles. React. Funct. Polym..

[2] Lalwani R., Desai S. (2010). Sorption behavior of biodegradable polyurethanes with carbohydrate crosslinkers. J. Appl. Polym. Sci..

[3] Kostić M., Cakić S., Marinović-Cincović I.R.M., Nikolić L., Samaržija-Jovanović S. (2021). Synthesis and characterization of pH-sensitive Saccharide modified polyurethane hydrogels–Effect of polyol, crosslinker and acid chain extender. Adv. Technol..

[4] Real D.A., Bolaños K., Priotti J., Yutronic N., Kogan M.J., Sierpe R., Donoso-González O. (2021). Cyclodextrin-modified nanomaterials for drug delivery: Classification and advances in controlled release and bioavailability. Pharmaceutics.

[5] de Araújo M.V.G., Vieira J.V.F., da Silva T.A., Kubota T., Barboza F.M., Farago P.V., Zawadzki S.F. (2012). Innovative cross-linked polyurethane networks based on cyclodextrins and polyethylene glycols: Inclusion capacity and potential use as controlled release carrier for nifedipine. Macromolecular Symposia.

[6] Gould S., Scott R.C. (2005). 2-Hydroxypropyl-β-cyclodextrin (HP-β-CD): A toxicology review. Food Chem. Toxicol..

[7] Cyclodextrins Used as Excipients. European Medicines Agency, Science, Medicines, Health, Committee for Human Medicinal Products CHMP/495747/2013, 9 October 2017, London, United Kingdom. https://www.ema.europa.eu/en/documents/scientific-guideline/questions-and-answers-cyclodextrins-used-excipients-medicinal-products-human-use_en.pdf.

[8] Omidian H., Akhzarmehr A., Gill E.J. (2025). Cyclodextrin–hydrogel hybrids in advanced drug delivery. Gels.

[9] Szalai B., Budai-Szucs M., Kovacs A., Berko S., Grof I., Deli M.A., Katona G., Balogh G.T., Jojart-Laczkovich O. (2024). The effect of mucoadhesive polymers on ocular permeation of thermoresponsive in situ gel containing dexamethasone-cyclodextrin complex. Int. J. Pharm..

[10] Mahajan H.S., Shah S.K., Surana S.J. (2011). Nasal in situ gel containing hydroxy propyl β-cyclodextrin inclusion complex of artemether: Development and in vitro evaluation. J. Incl. Phenom. Macrocycl. Chem..

[11] Iohara D., Okubo M., Anraku M., Uramatsu S., Shimamoto T., Uekama K., Hirayama F. (2017). Hydrophobically modified polymer/alpha-cyclodextrin thermoresponsive hydrogels for use in ocular drug delivery. Mol. Pharm..

[12] Helal D.A., Osama A., El-Nabarawi M.A., Teaima M.H., Al-Samadi I.E.I. (2025). Dual-action of clotrimazole loaded− nanosponges vaginal gel for spermicidal action and treatment of vaginal candidiasis: Optimization, in-vitro, ex-vivo, and in-vivo experiments. Int. J. Pharm..

[13] Naeem A., Yu C., Zhu W., Zang Z., Guan Y. (2023). Study of hydroxypropyl beta-cyclodextrin and puerarin inclusion complexes encapsulated in sodium alginate-grafted 2-acrylamido-2-methyl-1-propane sulfonic acid hydrogels for oral controlled drug delivery. Gels.

[14] Khalid F.M., Ijaz M., Mahmood A., Waqas M.K., Hussain T., Asim M.H., Ahmad N., Arshad S., Rehman M.U., Nazir I. (2023). Mucoadhesive, Fluconazole-loaded nanogels complexed with sulfhydryl-beta-cyclodextrin for oral thrush treatment. AAPS PharmSciTech.

[15] Becheri A., Lo Nostro P., Ninham B.W., Baglioni P. (2003). The curious world of polypseudorotaxanes: Cyclodextrins as probes of water structure. J. Phys. Chem. B.

[16] Diaconu A.-D., Logigan C.-L., Peptu C.A., Ibanescu C., Harabagiu V., Peptu C. (2023). Polyurethane degradable hydrogels based on cyclodextrin-oligocaprolactone derivatives. Gels.

[17] Harada A., Kamachi M. (1990). Complex formation between cyclodextrin and poly(propylene glycol). J. Chem. Soc. Chem. Commun..

[18] Harada A., Kamachi M. (1990). Complex formation between poly(ethylene glycol) and α-cyclodextrin. Macromolecules.

[19] Hoti G., Bajwa N., Caldera F., Singh P.A., Hussein I., Cecone C., Matencio A., Spagnolo R., Argenziano M., Cavalli R. (2025). Cyclodextrin-based therapeutics delivery systems: A review of current clinical trials. Curr. Res. Pharmacol. Drug Discov..

[20] Peng K., Chen C., Pan W., Liu W., Wang Z., Zhu L. (2016). Preparation and properties of β-cyclodextrin/4, 4′-diphenylmethane diisocyanate/polyethylene glycol (β-CD/MDI/PEG) crosslinking copolymers as polymeric solid–solid phase change materials. Sol. Energy Mater. Sol. Cells.

[21] Khatter N.J., Khan M.A. (2025). Clotrimazole. StatPearls.

[22] Mycelex^®^ (clotrimazole) Troche for Topical Oral Administration. https://s3-us-west-2.amazonaws.com/drugbank/fda_labels/DB00257.pdf?1265922797.

[23] Blokhina S., Sharapova A., Ol’khovich M., Perlovich G. (2019). Experimental solubility of clotrimazole and some thermodynamic aspects of dissolution in different solvents. Thermochim. Acta.

[24] Cakić S., Ristić I., Holló B., Nikolić V., Nikolić N., Rakić S., Ilić-Stojanović S. (2025). Thermoanalytical studies on cross-linked polyurethane networks: Effect of polyol molecular weight and structure of cyclodextrins. Polym. Bull..

[25] Cakić S., Nikolić V., Ristić I., Nikolić N., Ilić-Stojanović S. (2025). Thermoanalytical studies on complexes of clotrimazole with polyurethane networks based on cyclodextrins and polyethylene glycols. Book of abstracts XVI International Symposium Novel Technologies and Sustainable Development.

[26] Solanki A., Thakore S. (2015). Cellulose crosslinked pH-responsive polyurethanes for drug delivery: α-hydroxy acids as drug release modifiers. Int. J. Biol. Macromol..

[27] Nematpour N., Moradipour P., Zangeneh M.M., Arkan E., Abdoli M., Behbood L. (2020). The application of nanomaterial science in the formulation a novel antibiotic: Assessment of the antifungal properties of mucoadhesive clotrimazole loaded nanofiber versus vaginal films. Mater. Sci. Eng. C.

[28] Mohamed M.H., Wilson L.D., Headley J.V., Peru K.M. (2008). Novel materials for environmental remediation of tailing pond waters containing naphthenic acids. Process Saf. Environ. Prot..

[29] Bilensoy E., Abdur Rouf M., Vural I., Šen M., Atilla Hincal A. (2006). Mucoadhesive, thermosensitive, prolonged-release vaginal gel for clotrimazole: β-cyclodextrin complex. AAPS PharmSciTech.

[30] Mohammed N.N., Pandey P., Khan N.S., Elokely K.M., Liu H., Doerksen R.J., Repka M.A. (2016). Clotrimazole–cyclodextrin based approach for the management and treatment of Candidiasis—A formulation and chemistry-based evaluation. Pharm. Dev. Technol..

[31] Wang T.L., Hsieh T.H. (1997). Effect of polyol structure and molecular weight on the thermal stability of segmented poly(urethaneureas). Polym. Degrad. Stab..

[32] Taneri F., Güneri T., Aigner Z., Berkesi O., Kata M. (2004). Thermoanalytical studies on complexes of clotrimazole with cyclodextrins. J. Therm. Anal. Calorim..

[33] Solanki A., Mehta J., Thakore S. (2014). Structure–property relationships and biocompatibility of carbohydrate crosslinked polyurethanes. Carbohydr. Polym..

[34] Wang H., Xu J., Hu J., Hang G., Zhang T., Zheng S. (2023). Reprocessing and shape recovery of polyurethane enabled by crosslinking with poly (β-cyclodextrin) via host-guest interactions. Polymer.

[35] Lee Y.M., Lee J.C., Kim B.K. (1994). Effect of soft segment length on the properties of polyurethane anionomer dispersion. Polymer.

[36] Kosecka-Judin E., Wesolowski M., Paukszta D. (2012). Pattern recognition methods as supplementary techniques for identification of salicylamide—Cyclodextrins inclusion complexes. Cent. Eur. J. Chem..

[37] Xie A., Zhang M., Inoue S.I. (2016). Influence of β-cyclodextrin on morphologies and chemical, thermal, and mechanical properties of non-chain extended polyurethane elastomers. J. Polym. Res..

[38] Xie A., Zhang M., Inoue S.I. (2016). Influence of diisocyanate on polyurethane elastomers which crosslinked by β-cyclodextrin. Open J. Org. Polym. Mater..

[39] Chen Y., Xie A., Zhang M., Inoue S.I. (2017). Influences of polyol on the chemical, thermal, and mechanical properties of polyurethane elastomers crosslinked by β-cyclodextrin. Open J. Org. Polym. Mater..

[40] Balakrishnan P., Song C.K., Cho H.J., Yang S.G., Kim D.D., Yong C.S., Choi H.G. (2012). Inclusion complex effect on the bioavailability of clotrimazole from poloxamer-based solid suppository. Arch. Pharm. Res..

[41] Gaurav C., Nikhil G., Deepti S., Kalra S., Goutam R., Amit G.K. (2015). Albumin stabilized silver nanoparticles–clotrimazole β-cyclodextrin hybrid nanocomposite for enriched anti-fungal activity in normal and drug resistant Candida cells. RSC Adv..

[42] Hesari Z., Emmamzadehhashemi M.S.B., Aboutaleb E. (2023). Tragacanth and xanthan gum natural polymers for formulation of clotrimazole mucoadhesive gel. Acta Sci. Health Sci..

[43] Yakupova L.R., Skuredina A.A., Markov P.O., Le-Deygen I.M., Kudryashova E.V. (2023). Cyclodextrin Polymers as a Promising Drug Carriers for Stabilization of Meropenem Solutions. Appl. Sci..

[44] Pereira E.D., da Silva Dutra L., Paiva T.F., de Almeida Carvalho L.L., Rocha H.V.A., Pinto J.C. (2023). In Vitro Release and In Vivo pharmacokinetics of praziquantel loaded in different polymer particles. Materials.

[45] Kareem F., Bhayo A.M., Imran M., Shah M.R., Khan K.M., Malik M.I. (2019). Enhanced therapeutic efficacy of clotrimazole by delivery through poly(ethylene oxide)-block-poly(ε-caprolactone) copolymer-based micelles. J. Appl. Polym. Sci..

[46] Gabr M.M., Mortada S.M., Sallam M.A. (2018). Carboxylate cross-linked cyclodextrin: A nanoporous scaffold for enhancement of rosuvastatin oral bioavailability. Eur. J. Pharm. Sci..

[47] Yallapu M.M., Gupta B.K., Jaggi M., Chauhan S.C. (2010). Fabrication of curcumin encapsulated PLGA nanoparticles for improved therapeutic effects in metastatic cancer cells. J. Colloid Interface Sci..

[48] Stancu A.I., Mititelu M., Ficai A., Ditu L.-M., Buleandră M., Badea I.A., Pincu E., Stoian M.C., Brîncoveanu O., Boldeiu A. (2025). Comparative evaluation of β-cyclodextrin inclusion complexes with eugenol, eucalyptol, and clove essential oil: Characterisation and antimicrobial activity assessment for pharmaceutical applications. Pharmaceutics.

[49] Santos S.S., Lorenzoni A., Ferreira L.M., Mattiazzi J., Adams A.I., Denardi L.B., Alves S.H., Schaffazick S.R., Cruz L. (2013). Clotrimazole-loaded Eudragit^®^ RS100 nanocapsules: Preparation, characterization and in vitro evaluation of antifungal activity against Candida species. Mater. Sci. Eng. C.

[50] Sharma G., Jain S., Tiwary A.K., Kaur G. (2006). Once daily bioadhesive vaginal clotrimazole tablets: Design and evaluation. Acta Pharm..

[51] Balata G., Mahdi M., Bakera R.A. (2011). Improvement of solubility and dissolution properties of clotrimazole by solid dispersions and inclusion complexes. Indian J. Pharm. Sci..

[52] Rowe R.C., Sheskey P.J., Weller P.J. (2006). Handbook of Pharmaceutical Excipients.

[53] Trucillo P. (2022). Drug Carriers: A Review on the most used mathematical models for drug release. Processes.

[54] Gouda R., Baishya H., Qing Z. (2017). Application of mathematical models in drug release kinetics of carbidopa and levodopa ER tablets. J. Dev. Drugs.

[55] Costa P., Lobo J.M.S. (2001). Modeling and comparison of dissolution profiles. Eur. J. Pharm. Sci..

[56] Elmas A., Akyüz G., Bergal A., Andaç M., Andaç Ö. (2020). Mathematical modelling of drug release. Res. Eng. Struct. Mat..

[57] Zhu W., Long J., Shi M. (2023). Release Kinetics Model Fitting of Drugs with Different Structures from Viscose Fabric. Materials.

[58] Barati Farimani A., Aluru N.R. (2011). Spatial diffusion of water in carbon nanotubes: From Fickian to ballistic motion. J. Phys. Chem. B.

